# Anomalous Left Circumflex Artery Arising From the Right Coronary Artery: A Cadaveric Case Report and Review of the Literature

**DOI:** 10.7759/cureus.49380

**Published:** 2023-11-25

**Authors:** Benjamin R Mann, Aaron W Beger

**Affiliations:** 1 Biomedical Sciences, Edward Via College of Osteopathic Medicine, Blacksburg, USA; 2 Anatomical Sciences, Edward Via College of Osteopathic Medicine, Blacksburg, USA

**Keywords:** cardiac anatomy, right coronary artery (rca), cadaver case report, coronary vessel anomaly, anamolous left circumflex artery

## Abstract

Coronary artery anomalies are relatively rare in the general population; however, they remain clinically significant due to their varying effects on cardiovascular function and diagnostic and treatment outcomes. Here is described an anomalous left circumflex artery (ALCx) discovered during routine dissection of a 76-year-old female anatomical donor. The ALCx was seen arising from shared ostia with the right coronary artery and conus artery from the right aortic sinus of Valsalva, giving off the left atrial branch along its retroaortic course before reaching the left aspect of the coronary sulcus. The left coronary artery took a traditional course, arising from the left aortic sinus of Valsalva before traveling in the anterior interventricular sulcus. A review of the literature was conducted to determine the incidence of ALCx and elucidate any associated clinical considerations. Though relatively rare, clinical awareness is necessary as evidence indicates ALCx, particularly the retroaortic portion, may be more prone to atherosclerosis, intimal proliferation, luminal occlusion, and increased ratio of necrotic core in atherosclerotic plaques. Imaging studies, including the aortic root sign on left ventriculography, can aid in the identification of ALCx. Awareness of ALCx and its potential influence on cardiac health is critical for the avoidance of diagnostic errors and adverse treatment outcomes. Through this case report, we seek to present the current evidence outlining the incidence of ALCx, as well as the literature surrounding its clinical implications.

## Introduction

Coronary artery anomalies (CAA) are relatively rare in the general population, and while most are discovered incidentally or remain undetected throughout a person's life, some anomalies can have potentially serious complications. It has been shown that certain CAAs can have significant effects on coronary hemodynamics and have been implicated as a source of sudden cardiac death, and some authors have suggested that others may be more prone to the development of atherosclerotic disease [1,2]. Additionally, CAAs can further complicate patients undergoing coronary angiography or cardiac surgery if the physician is unaware of the existence of the anomaly [1,3-6]. For these reasons, it is of the utmost importance for clinicians to be aware of the presence of these anomalies in their patients.

In normal embryological development, the coronary arteries develop within the epicardial interventricular and atrioventricular grooves, with the proximal ends of the arteries growing into their respective aortic sinuses; however, the exact process by which coronary vessels are formed is still being researched [2,7]. The coronary arteries are normally distributed with the right coronary artery (RCA) arising from the right sinus of Valsalva (RSV) and the left coronary artery (LCA) arising from the left sinus of Valsalva (LSV). The RCA typically descends from its origin through the right atrioventricular sulcus to reach the right margin of the heart, from there it courses along the diaphragmatic surface and base of the heart [7]. Along its course, the RCA typically gives rise to the conus artery, sinus node artery, right marginal branch, atrioventricular nodal branch, and posterior interventricular branch [7]. The LCA has a short course and quickly bifurcates into the left anterior descending (LAD) artery and the left circumflex (LCx) artery. The LAD continues in the anterior interventricular sulcus towards the apex of the heart and gives rise to diagonal and septal branches [7]. The LCx continues towards the left side of the heart in the coronary sulcus and typically gives rise to the left marginal artery, the posterolateral branch, and the obtuse marginal branch before terminating on the diaphragmatic surface of the heart [7].

The overall prevalence of CAA has been reported to be around 1-1.3% [4,5,8,9]. Several CAA have been described, each with its own respective incidence and clinical implications. These anomalies can be generally divided into benign and malignant. Benign CAA has relatively little effect on cardiovascular function and includes a separate origin of LCx and LAD, absent LCx, origin of LCx from RCA or RSV, origin of LCA/RCA from the posterior aortic sinus, origin from ascending aorta, and intercoronary communication [4]. Malignant CAA is more likely to cause cardiovascular dysfunction leading to angina, myocardial infarction, ventricular tachycardia, or cardiac arrest, and includes ectopic left coronary from the pulmonary artery (Bland-White-Garland syndrome), LCA from the RSV, RCA from the LSV, a single coronary artery, and coronary fistulae [4]. Benign coronary anomalies comprised 81% of anomalies discovered by Yamanaka and Hobbs [4].

In our report, we describe a case of an anomalous left circumflex artery (ALCx) arising as a branch of the RCA discovered during routine dissection of a formalin-fixed anatomical donor by first-year medical students. Prior studies report varying incidence rates and a range of clinical factors that may be precipitated by the presence of ALCx, including atherosclerosis, intimal proliferation, luminal occlusion, and increased ratio of necrotic core in atherosclerotic plaques. Thus, we have conducted a literature review to elucidate the incidence of ALCx as well as its clinical implications.

## Case presentation

**Figure 1:**
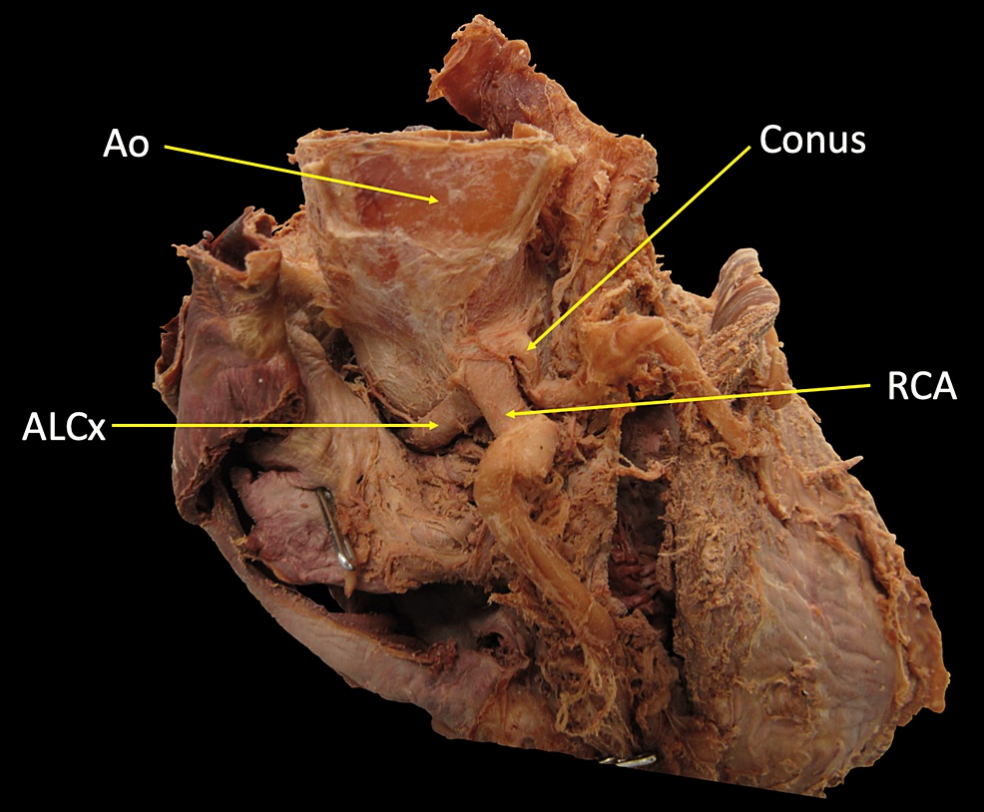
Anterior view of the heart depicting the origin of ALCx from shared ostia with RCA and conus artery ALCx: Anomalous left circumflex artery; Ao: Aorta; RCA: Right coronary artery

This study was reviewed and received ethical approval by the Institutional Review Board Committee of Edward Via College of Osteopathic Medicine (VCOM IRB Record #2022-067). A formalin-fixed donor willed to Edward Via College of Osteopathic Medicine via the Virginia State Anatomical Board was found to have an ALCx arising from a shared ostia with the RCA and conus artery during routine dissection by first-year medical students (Figure 1). The donor was a 76-year-old female with a history of Parkinson's disease and whose cause of death was non-cardiac related. No other cardiac anatomical abnormalities were noted.

**Figure 2:**
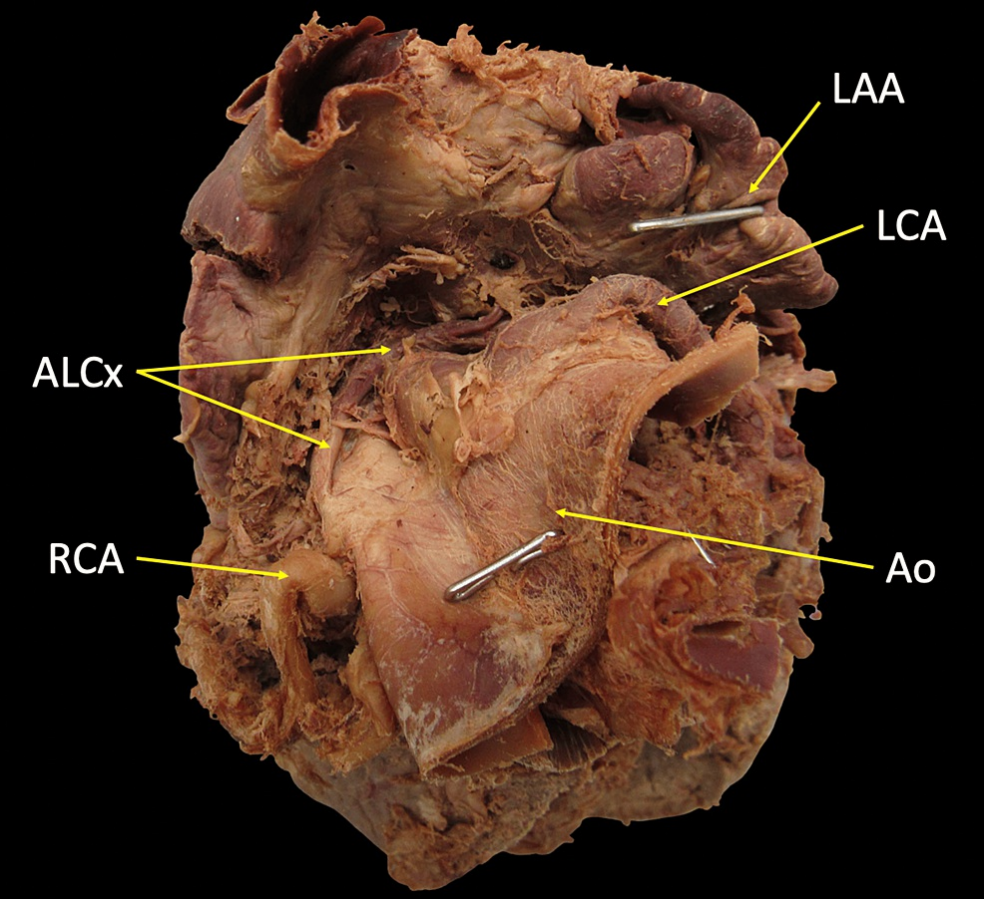
Superior view of the heart with aorta retracted anteriorly to depict the retroaortic course of ALCx ALCx: Anomalous left circumflex artery; Ao: Aorta; LAA: Left atrium; LCA: Left coronary artery; RCA: Right coronary artery

The ALCx demonstrated a retro-aortic course as it traveled between the root of the aorta and the left atrium (Figure 2).

**Figure 3:**
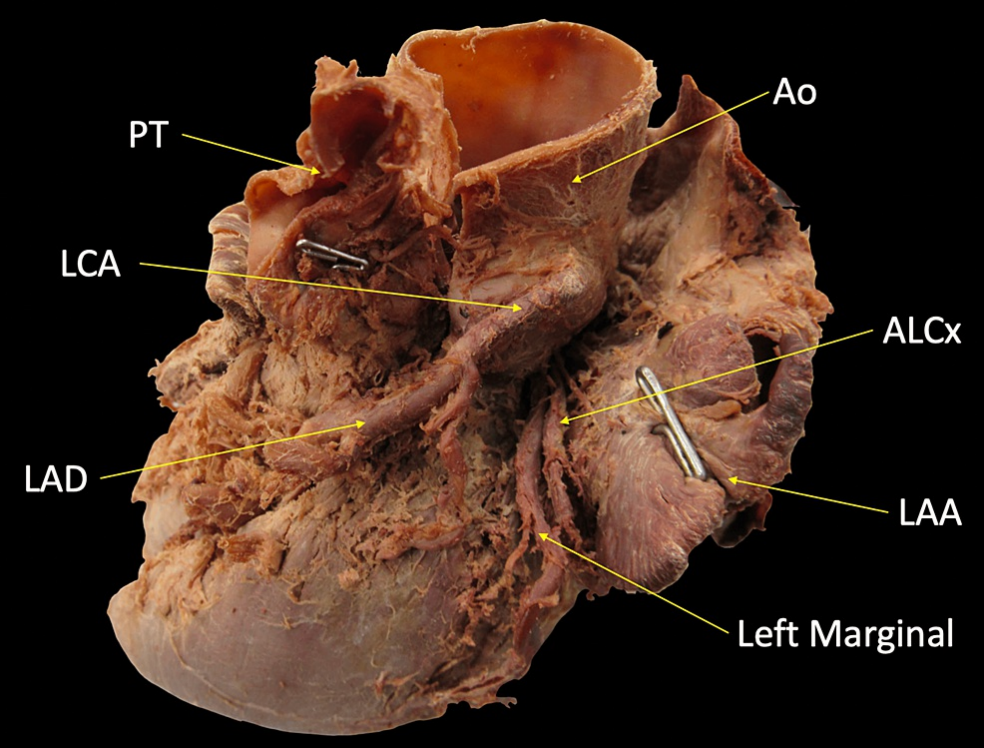
View of the left margin of the heart with the left atrium retracted to reveal a course of ALCx in the left aspect of the coronary sulcus ALCx: Anomalous left circumflex artery; Ao: Aorta; LAA: Left atrium; LAD: Left anterior descending artery; LCA: Left coronary artery; PT: Pulmonary trunk

The ALCx then emerged in the left aspect of the coronary sulcus where it gave off the left marginal branch (Figure 3). The ALCx measured 11.83mm in length from the RCA to the origin of the left marginal artery and had a maximal diameter of 3.02mm.

## Discussion

In our report, we discovered an ALCx arising as a branch of the RCA on a heart belonging to a 79-year-old formalin-fixed donor. While most authors agree that this is one of the most common CAAs observed, many have suggested that the true incidence of this anomaly is difficult to identify [3]. The incidence rates of ALCx reported in the literature are summarized in Table 1, and range from 0.15% to 0.67%, though rates as high as 16.2% have been observed in subjects with transposition of the great arteries [1,3]. Page et al. reviewed the angiography reports of 2,996 patients seen at their facility and discovered the ALCx in 20 patients, giving this anomaly an incidence of 0.67% for this study, and is the most common incidence rate cited in the recent literature [3]. Yamanaka and Hobbs conducted a similar study in which patients undergoing coronary angiography were screened for isolated CAA. This study included a significantly larger sample of patients with an n=126,595. Of these patients, 1,686 were identified as having CAAs, 467 of which were ALCx, giving an incidence rate of 0.37% for this study [4].

**Table 1:** A summary of the incidence of anomalous left circumflex artery (ALCx) arising from the right coronary artery (RCA) and relevant clinical observations ALCx: Anomalous left circumflex artery; CAA: Coronary artery anomaly; CAD: Coronary artery disease; CCTA: Cardiac computed tomography angiography; ECG: Electrocardiogram; LAS: Left aortic sinus; LCA: Left coronary artery; PCI: Percutaneous coronary intervention; RAS: Right aortic sinus; RCA: Right coronary artery; TGA: Transposition of the great arteries

Reference	Incidence of ALCx arising from RCA	Clinical summary
West et al., 2006 [1]	12/8,022 (0.15%)	A review of coronary angiograms revealed three distinct anatomical variants of the ALCx ostium: type I: separate ostia for RCA and ALCx within RAS (6/8,022; 0.074%) type II: common/adjacent ostia in RAS (3/8022; 0.037%) type III: ALCx arising as a branch of the proximal RCA (3/8,022; 0.037%).
Page et al., 1974 [3]	20/2,996 (0.67%)	ALCx was demonstrated via the aortic root sign and a sign of nonperfused myocardium. The aortic root sign is when a profile view of the ALCx can be seen as it courses posteriorly to the RAS during left ventriculography in a right anterior oblique projection. This sign was demonstrated in 19/20 patients, the only exception being due to complete occlusion of the ALCx. A sign of nonperfused myocardium is observed as an avascular area seen in the posterior lateral left ventricular myocardium during selective opacification of the LCA, which suggests the presence of ALCx.
Yamanaka and Hobbs, 1990 [4]	467/126,595 (0.37%)	CAA was identified in 1,686/126,595 (1.33%) patients. 1,461 of those (87%) were identified as having anomalies of artery origin or distribution, while 225 (13%) had coronary fistulae. ALCx arising from the RAS or as a branch of the RCA was identified in 467 (0.37%) of patients.
Szymczyk et al., 2014 [8]	4/726 (0.55%)	The results of ECG-gated CCTA in 726 consecutive patients were analyzed retrospectively. The overall incidence of coronary artery anomalies was 8/726 (1.1%). The most common anomaly was an ALCx from the RAS with a retroaortic course (0.55%) followed by the origin of RCA from the LAS (0.3%).
Mohsen et al., 2013 [10]	18/5205 (0.34%)	A total of 18 ALCx were identified out of 5,205 angiograms. CAD of any severity was present in 16/18 (89%) patients. 1/18 (5.5%) had obstructive CAD isolated to the ALCx. Within the studied cohort, the number of patients with one of the three variants was determined to be: type I: 1/18 (6%) type II: 13/18 (72%) type III: 4/18 (22%).
Yeşilyurt et al., 2017 [11]	9/2,973 (0.30%)	Of 2973 patients undergoing CCTA, 79 (2.65%) were diagnosed with CAA. The frequency of CAA in this study included: RCA originating from the LAS (0.50%), left circumflex originating from the RAS (0.10%), LCA originating from the RS (0.23%), LCA and RCA originating from the pulmonary artery (0.03%), and left circumflex originating from the RCA (0.20%).
Ratti et al., 2023 [12]	18/12,593 (0.14%)	Out of 12,593 CCTAs performed, 92 (0.71%) of patients were identified as having CAA. Of these, 18 (0.14%) were identified as having an ALCx arising from the RAS.
Randhawa et al., 2017 [13]	1/150 (0.67%)	In a study of 150 cadaveric hearts, one ALCx was found in a 45-year-old female. The artery arose along with the RCA from a common ostium in the RAS and depicted a retroaortic course.
Moll et al., 2017 [14]	116/715 (16.2%) of TGA subjects	All patients with TGA who underwent an arterial switch operation between 1991 and 2015 were consecutively enrolled in this study. The study identified 715 patients with isolated and complex TGA. CAA was present in 241 (33.7%) patients and was significantly correlated with the side-by-side configuration of the great arteries. The most frequent anomaly, which constituted approximately half of the variants described, was ALCx from the RCA. This anomaly was not shown to affect post-operative outcomes.

Subsequent studies continue to report variable results. West et al. identified 12 patients with an ALCx out of 8,022 undergoing coronary angiography for percutaneous coronary intervention (PCI) with an incidence of 0.15% [1]. This study brings about the classification of this anomaly into type I, type II, and type III based on the description of the possible variants seen by Page et al. Type I is defined as the ALCx arising from a separate ostium within the RSV, type II has a common/adjacent ostium in the right sinus, and in type III the ALCx arises as a proximal branch of the RCA [1]. Mohsen et al. also reviewed coronary angiograms for the presence of an ALCx, with additional investigation into the relative incidence of each type of ALCx. About 5,205 angiograms were reviewed during the study period, identifying 18 patients with ALCx, an incidence rate of 0.34% [10]. Out of those 18, one was identified as having a type I variant (6%), 13 had type II (72%), and 4 had type III (22%) [10]. Though this is a small sample of patients, this is the first study to our knowledge that suggests ALCx with a shared ostium with the RCA is the most prevalent of the three types.

Studies by Szymczyk et al., Yeşilyurt al., and Ratti et al. each used coronary computed tomography angiography (CCTA) as their imaging modality for identifying CAAs; however, again each study yielded different incidence rates for ALCx. Szymczyk et al. retrospectively reviewed the CCTA studies of 726 patients, identifying 4 patients with ALCx (0.55%) [8]. Yesilyurt et al. reviewed 2,973 CCTAs, 9 of which were identified as either having the ALCx arising from the RSV or as a proximal branch of the RCA (0.30%) [11]. In the study by Ratti et al., 12,593 CCTAs were analyzed with 18 being identified as having ALCx (0.14%) [12]. The only study that replicated the incidence rate reported by Page et al. was a survey of cadaveric hearts performed by Randhawa et al., in which one out of 150 cadaveric hearts were found to have an ALCx (0.67%) [13]. The generally lower incidence rates observed via CCTA may indicate an inability to detect all cases of CAA, though cadaveric studies with larger sample sizes would be needed to thoroughly scrutinize CCTA as a mode of CAA detection. Finally, a study of patients with transposition of the great arteries (TGA) by Moll et al. found that out of 715 patients with TGA, 116 were shown to have an ALCx (16.2%) [14]. This finding suggests that CAAs, including ALCx, tend to be more common in patients with congenital anomalies of the great vessels than in the general population.

In addition to the widely differing incidence rates reported for the ALCx, there is also a debate on the clinical implications of this anomaly. The ALCx is primarily regarded as a benign anomaly; however, authors have suggested that the ALCx may be prone to atherosclerosis, particularly in the retroaortic portion of the vessel. West et al. found significant atherosclerotic disease in the ALCx of 73% of patients in their study, while only 47% had significant disease in the other major coronary vessels [1]. This finding suggests that the existence of atherosclerotic disease in the ALCx is not able to predict the presence of disease in other vessels, and thus the ALCx is more prone to disease [1]. Obaid et al., using intravascular ultrasound, found that atherosclerotic lesions in the ALCx contained a higher percentage of necrotic core than those in the RCA (53% vs 32%, respectively) in a 45-year-old male presenting for chest pain [15]. The ratio of necrotic core to dense calcium was also noted to be twice as high in the ALCx as compared to the RCA (5.4 vs 2.7, respectively), a factor that has previously been associated with high-risk plaques [15]. Additionally, Randhawa et al. found a greater degree of intimal proliferation and luminal occlusion in ALCx as compared to other coronary arteries [13]. However, a study of coronary angiograms of patients undergoing PCI by Mohsen et al. suggests that the ALCx is not more prone to atherosclerotic disease. In this study, seven out of 18 patients with ALCx were found to have obstructive atherosclerotic disease in the anomalous artery, but significant disease was also found in both the LAD and RCA [10]. Studies by Ratti et al. and Fiorilli et al. also refute the claim that the ALCx is more prone to atherosclerotic disease [12,16].

While many aspects of the ALCx are still debated in the literature, one thing that is agreed upon is the importance of being aware of the anomaly during coronary angiography or cardiac surgery. Not only can the lack of knowledge of the anomaly lead to diagnostic errors but may also affect treatment outcomes. An example of this is a case reported by Mosenska et al. where a 23-year-old male experienced an inferolateral myocardial infarction during surgery for a patent ductus arteriosus after cardioplegia was improperly administered due to a lack of knowledge of the patient ALCx [6]. Fukunaga et al. also presented a case in which an ALCx was discovered intraoperatively during an aortic valve replacement. The close association of this anomaly to the aortic root required a remodeling root replacement to be performed in order to avoid injury to or compression of the ALCX [17].

## Conclusions

Here we reported a cadaveric heart with an ALCx arising as a proximal branch of the RCA, which then took a retroaortic course to supply the left margin of the heart. While this has been regarded as one of the most common CAAs, there has been variation in the literature regarding the incidence of this anomaly and whether it can truly be regarded as “benign.” Nonetheless, it is important that cardiologists and cardiac surgeons be aware of this anomaly when it exists, as lack of awareness can affect diagnosis and lead to adverse treatment outcomes. Thus, physicians should remain suspicious of this anomaly, keeping in mind the relative incidence in the population.
